# Evaluating the expected effects of disclosure of patient safety incidents using hypothetical cases in Korea

**DOI:** 10.1371/journal.pone.0199017

**Published:** 2018-06-14

**Authors:** Minsu Ock, Eun Young Choi, Min-Woo Jo, Sang-il Lee

**Affiliations:** 1 Department of Preventive Medicine, Ulsan University Hospital, University of Ulsan College of Medicine, Ulsan, Republic of Korea; 2 Department of Preventive Medicine, University of Ulsan College of Medicine, Seoul, Republic of Korea; Medical University Graz, AUSTRIA

## Abstract

To introduce disclosure of patient safety incidents (DPSI) into a specific country, evidence of the effectiveness of DPSI is essential. Since such a disclosure policy has not been adopted in South Korea, hypothetical cases can be used to measure the effectiveness of DPSI. We evaluated the effectiveness of DPSI using hypothetical cases in a survey with a sample of the Korean general public. We used 8 hypothetical cases reflecting 3 conditions: the clarity of medical errors, the severity of harm, and conducting DPSI. Face-to-face interviews with 700 people using structured questionnaires were conducted. Participants were asked to read each hypothetical case and give remarks on the following: their judgment of a situation as a medical error and of the requirement for an apology, the willingness to revisit or recommend physicians, the intention to file a medical lawsuit and commence criminal proceedings against physicians, the level of trust in physicians, and the expected amount of compensation. The results indicated favorable findings in support of DPSI; DPSI reduced the likelihood of perceiving a situation as a medical error, promoted willingness to revisit and recommend physicians, and discouraged the intention to file a medical lawsuit and take commence criminal proceedings against physicians. Furthermore, DPSI increased patients’ trust scores in physicians and reduced the expected amount of compensation. The general public had positive attitudes towards DPSI in South Korea. This result provides empirical evidence for reducing the psychological burden that the introduction of DPSI may have on health professionals.

## Introduction

When patient safety incidents occur, managing them is an important issue. In particular, policies and interventions that are adopted in order to respond to patient safety incidents are intended to manage such incidents and minimize any additional harm [1]. Tort law, no-fault liability for compensation, the alternative dispute resolution system, and disclosure of patient safety incidents (DPSI) are examples of legislations and institutions involved in the handling of patient safety incidents. Among them, DPSI is more advanced than tort law or the alternative dispute resolution system because it can prevent potential medical disputes in advance [2]. DPSI is defined as follows [3]: “When a patient safety incident occurs, medical professionals preemptively explain the incident to the patients and their caregivers, express sympathy and regret for the incident, deliver an apology and compensation appropriately if needed, and promise to prevent recurrence.”

Many countries and organizations have either implemented DPSI as their standards of accreditations [4, 5] or have developed guidelines to facilitate it [6, 7]. Such willingness to adopt DPSI stems not only from its ethical background [8–10], but also from its widely known benefits in reducing medical law suits and court costs [11]. However, studies on DPSI are confined to some Western countries, and evidence collected from non-Western countries, including South Korea (hereinafter Korea), is scarce [12, 13]. In order to introduce DPSI in a specific country, empirical evidence of the effectiveness of the DPSI is essential [14, 15]. In Korea, studies have been performed to analyze perceptions of DPSI using both qualitative [3] and quantitative [16, 17] methods. However, efforts to assess the effectiveness of DPSI are unprecedented in Korea, because DPSI has not been implemented in the real healthcare setting.

In this study, we evaluated the effectiveness of DPSI using hypothetical cases in a survey with a sample of the general public representing the Korean population. Since the disclosure policy has not been adopted in Korea, hypothetical cases can be alternatively used to measure the effectiveness of DPSI [18–20]. The results of this study are expected to serve as supporting evidence for the introduction of DPSI in Korea.

## Materials and methods

### Development of survey questions

We surveyed the general public in Korea using hypothetical cases and evaluated the effectiveness of DPSI. Previous studies that investigated the effectiveness of DPSI using hypothetical cases were assessed using a systematic review [11]. Questionnaires were drafted and modified based on advice from an expert in patient safety. In addition, further revisions were made to reflect findings from cognitive debriefing interviews with 2 lay persons.

The socio-demographic factors of survey participants were identified and included residential area, gender, age, level of education, religion, and whether they have physicians or nurses in their family. The scope of family was defined as participants’ parents, siblings, and children.

The 8 hypothetical cases (Table 1) were adapted from previous studies [3]. The hypothetical cases reflected the clarity of medical errors (unclear or clear), the severity of harm (minor or major), and conducting DPSI (full disclosure or no disclosure). The entire contents of the cases are available in Supporting Information File (S1 File). The distinction between full and partial DPSI was made according to the level of accomplishment of the following key factors: providing explanations, expressing sympathy, promising thorough investigation, apologizing, guaranteeing reasonable amount of compensation, and assuring prevention of recurrence [21]. Full DPSI was assumed in our survey.

**Table 1:** Hypothetical cases and survey factors.

				Clarity of medical errors	
				Unclear	Clear
Level of harm	Minor	Disclosure of patient safety incidents	Full disclosure	Case 1	Case 5
			No disclosure	Case 2	Case 6
	Major	Disclosure of patient safety incidents	Full disclosure	Case 3	Case 7
			No disclosure	Case 4	Case 8

Participants were asked to read the hypothetical cases and give remarks on their judgment of a situation as a medical error and the need for an apology, the willingness to revisit or recommend physicians from the hypothetical cases, and the intention to file a medical lawsuit and take commence criminal proceedings against physicians. For these items, a 4-point Likert scale (strongly disagree, disagree, agree, and strongly agree) was adopted. Furthermore, the trust score of the hypothetical physicians was measured on a scale from 1 point (worst) to 10 points (best). Finally, participants were asked to decide the expected amount of compensation and write 0 if they did not feel it was necessary.

In cases where medical errors are obvious (from cases 5–8) the questionnaire item on the need for an apology was omitted because most participants were expected to give positive answers. Therefore, this item was included only from case 1 to case 4. Since the order in which hypothetical cases are presented could possibly influence a participant’ response, 2 different questionnaire layouts were designed: type A, which presents DPSI first, and type B, which presents non-DPSI first.

### Conducting survey

The survey was conducted by Gallup Korea. Face-to-face interviews using structured questionnaires were carried out. A total of 700 members of the general public representing the Korean population were selected using quota sampling, where gender, age, and residential region (except for Je-ju Island) served as classification criteria for the quota. The population was defined as Korean citizens registered in the Ministry of Government Administration and Home Affairs by June 2015.

A single session was held to train interviewers on contents of the survey; the session lasted for approximately 90 minutes. The survey was conducted for approximately 1 month, from July to August 2015. Each interviewer alternated between 2 survey layouts (types A and B). Explanations were given to participants to familiarize them with terms related to patient safety, such as patient safety, patient safety incident, adverse event, medical error, near miss, and DPSI. Visual aids were used to assist participants with poor eyesight.

### Data analysis

In order to evaluate the effectiveness of DPSI in hypothetical cases, both multiple linear regression and logistic regression were used. When conducting linear regression, the trust score of the assumed physicians and the expected amount of compensation were used as dependent variables, and socio-demographic factors of participants (gender, age group, level of education, religion, physicians or nurses in the family, and experience with patient safety incidents) and variables related to the survey or cases (survey layouts, clarity of medical errors, severity of harm, and conducting DPSI) were included as independent variables. The expected amount of compensation in the hypothetical cases, especially in the case of minor harm, showed right-skewed distribution. Data transformation was performed before analysis. After adding 1 to all suggested amounts of compensation and converting them to natural logarithmic form, we performed further linear regression.

For logistic regression, judgment of a situation as a medical error and the need for an apology, willingness to revisit or recommend physicians from the hypothetical cases, and the intention to file a medical lawsuit and commence criminal proceedings against physicians were included as dependent variables. The response scale was re-categorized; “strongly disagree” and “disagree” were merged into “disagree,” while “agree” and “strongly agree” were merged into “agree.”

Stata/SE 13.1 and SPSS 21.0 were used to analyze the data. A significance level of 0.05 was used.

### Ethics statement

This study was approved by the Institutional Review Board of Asan Medical Center (2015–069). Prior to enrollment, we explained the objectives and processes of this study to the participants and obtained verbal informed consent from them. We obtained verbal informed consent rather than written consent, because this study presented no more than minimal risk of harm to participants and the only record linking the participants and the study would be the consent document.

## Results

### Characteristics of the participants

The survey response rate was 39.8%. Table 2 shows the socio-demographic characteristics of the participants. The mean age was 45.5 years (standard deviation, 14.5). The distribution of age and gender of survey participants were statistically insignificant compared with the national registration population data of the Ministry of Government Administration and Home Affairs in June 2015.

**Table 2:** Participants’ socio-demographic factors.

Variable		In this study		Registration of resident data^a^	*P*-value^b^
		N	%	%	
Age group (years)	19–29	125	17.9	17.7%	0.924
	30–39	131	18.7	18.5%	
	40–49	148	21.1	21.4%	
	50–59	139	19.9	19.8%	
	≥60	157	22.4	22.6%	
Gender	Man	348	49.7	49.6%	1.000
	Woman	352	50.3	50.4%	
Educational level	Elementary school or below	43	6.1	-	-
	Middle school	57	8.1	-	
	High school or attending college	495	70.7	-	
	College or above	105	15.0	-	
Religion	Yes	327	46.7	-	-
	No	373	53.3	-	
Physicians or nurses in the family	Yes	71	10.1	-	-
	No	629	89.9	-	

^a^The data are from the Ministry of the Interior as of June 2015.

^b^Chi-square test in SPSS 21.0

### Evaluation of patient safety incidents using hypothetical cases

The results of logistic regression revealed that the odds of judging the hypothetical cases as medical errors were 0.63 times lower (95% confidence interval (CI), 0.52–0.76) when DPSI was performed than when it was not (Table 3). However, for the necessity of an apology, the influence of DPSI was statistically insignificant (Table 3). The odds of willingness to revisit and recommend the same physician were 2.66 (95% CI: 2.35–3.01) and 2.47 times (95% CI: 2.14–2.85) higher, respectively, when patient safety incidents were disclosed than when they were not disclosed (Table 3). The odds of the intention to file a medical lawsuit were 0.41 times (95% CI: 0.35–0.47) lower and the odds of the intention to take criminal proceedings were 0.44 times (95% CI: 0.38–0.50) lower (Table 3) when the incidents were disclosed than when they were not disclosed.

**Table 3:** Estimated effects of disclosure of patient safety incidents by logistic regression.

	Judging a situation as a medical error			Necessity of an apology			Willingness to revisit			Willingness to recommend			Intention to file a lawsuit			Commence criminal proceedings		
	OR	95% CI		OR	95% CI		OR	95% CI		OR	95% CI		OR	95% CI		OR	95% CI	
		Lower	Upper		Lower	Upper		Lower	Upper		Lower	Upper		Lower	Upper		Lower	Upper
Gender																		
Men	Ref			Ref			Ref			Ref			Ref			Ref		
Women	0.86	0.70	1.04	0.96	0.70	1.33	1.05	0.93	1.19	1.09	0.95	1.26	**1.15**	**1.00**	**1.33**	0.99	0.86	1.13
Age group (years)																		
19–29	Ref			Ref			Ref			Ref			Ref			Ref		
30–39	**1.46**	**1.05**	**2.02**	0.93	0.53	1.64	**0.80**	**0.66**	**0.99**	0.82	0.64	1.04	0.96	0.77	1.21	0.90	0.73	1.12
40–49	1.03	0.76	1.39	**0.46**	**0.27**	**0.76**	0.96	0.78	1.17	1.04	0.82	1.30	**0.70**	**0.56**	**0.88**	0.86	0.69	1.06
50–59	1.07	0.77	1.50	0.72	0.40	1.31	1.05	0.84	1.30	0.99	0.77	1.27	**0.76**	**0.59**	**0.97**	0.98	0.78	1.24
≥60	1.09	0.77	1.55	0.62	0.34	1.14	0.90	0.72	1.13	0.88	0.68	1.14	0.89	0.69	1.14	0.99	0.78	1.26
Education level																		
College or below	Ref			Ref			Ref			Ref			Ref			Ref		
College graduate or above	0.93	0.74	1.17	0.70	0.48	1.02	0.94	0.81	1.09	0.93	0.79	1.11	1.07	0.90	1.26	1.15	0.98	1.34
Religion																		
No	Ref			Ref			Ref			Ref			Ref			Ref		
Yes	**1.33**	**1.09**	**1.63**	1.29	0.93	1.80	0.99	0.87	1.13	0.99	0.86	1.15	**1.26**	**1.09**	**1.45**	**1.19**	**1.03**	**1.36**
Physicians or nurses in the family																		
No	Ref			Ref			Ref			Ref			Ref			Ref		
Yes	0.86	0.63	1.17	0.82	0.50	1.35	**0.79**	**0.64**	**0.97**	1.03	0.81	1.30	0.81	0.64	1.01	**0.69**	**0.56**	**0.87**
Experiences of patient safety incidents																		
No	Ref			Ref			Ref			Ref			Ref			Ref		
Yes	**0.64**	**0.47**	**0.89**	1.12	0.61	2.03	**0.73**	**0.57**	**0.93**	0.77	0.58	1.02	0.98	0.76	1.27	0.84	0.66	1.08
Survey layout																		
Type A	Ref			Ref			Ref			Ref			Ref			Ref		
Type B	1.15	0.95	1.40	1.22	0.89	1.68	**0.64**	**0.57**	**0.73**	**0.56**	**0.49**	**0.65**	**0.64**	**0.56**	**0.73**	**0.69**	**0.60**	**0.78**
Clarity of medical errors																		
Unclear	Ref			-			Ref			Ref			Ref			Ref		
Clear	**3.87**	**3.10**	**4.83**	-	-	-	**0.73**	**0.64**	**0.82**	**0.52**	**0.45**	**0.60**	**2.07**	**1.80**	**2.38**	**1.81**	**1.59**	**2.06**
Level of harm																		
Minor	Ref			Ref			Ref			Ref			Ref			Ref		
Major	**3.48**	**2.80**	**4.32**	**4.80**	**3.24**	**7.11**	**0.21**	**0.19**	**0.24**	**0.20**	**0.17**	**0.24**	**23.08**	**19.86**	**26.81**	**13.21**	**11.43**	**15.26**
Disclosure of patient safety incidents																		
No disclosure	Ref			Ref			Ref			Ref			Ref			Ref		
Full disclosure	**0.63**	**0.52**	**0.76**	0.93	0.68	1.27	**2.66**	**2.35**	**3.01**	**2.47**	**2.14**	**2.85**	**0.41**	**0.35**	**0.47**	**0.44**	**0.38**	**0.50**

OR: odds ratio, CI: confidence interval

Some variables also showed statistically significant relationships in the logistic regression. For example, the odds of judging the hypothetical cases as medical errors were 3.87 (95% CI: 3.10–4.83) and 3.48 times (95% CI: 2.80–4.32) higher, when the clarity of medical errors was clear and major harm occurred than when the clarity of medical errors was unclear and minor harm occurred, respectively. There was a statistically significant relationship between the necessity of an apology and the level of harm. In particular, the odds of the intention to file a lawsuit and to commence criminal proceedings were 23.08 (95% CI: 19.86–26.81) and 13.21 times (95% CI: 11.43–15.26) higher, when major harm occurred than when minor harm occurred, respectively.

Next, the results of linear regression were checked to confirm the magnitude of the impact of DPSI on the trust score of assumed physicians and the expected amount of compensation. Trust scores were higher by 1.24 points (95% CI: 1.15–1.34) when patient safety incidents were disclosed than when they were not disclosed (Table 4). DPSI reduced the amount of expected compensation by 16,370,000 won (95% CI: -25,730,000–-7,000,000). When we used the natural log transformed value as an outcome variable, the expected amount of compensation was still smaller when patient safety incidents were disclosed than when they were not disclosed (Table 4).

**Table 4:** Estimated effects of disclosure of patient safety incidents using linear regression.

	Trust score of the physician			Expected amount of compensation (10,000 won)^a^		
	Coefficient	95% CI		Coefficient	95% CI	
		Lower	Upper		Lower	Upper
Gender						
Men	Ref			Ref		
Women	0.07	-0.03	0.17	**0.18**	**0.03**	**0.33**
Age						
19–29	Ref			Ref		
30–39	**-0.19**	**-0.35**	**-0.03**	0.20	-0.05	0.44
40–49	-0.13	-0.29	0.03	0.13	-0.11	0.38
50–59	0.00	-0.17	0.17	0.07	-0.20	0.33
≥60	-0.17	-0.35	0.01	-0.01	-0.28	0.26
Education level						
College or below	Ref			Ref		
College graduate or above	-0.08	-0.20	0.03	-0.07	-0.25	0.11
Religion						
No	Ref			Ref		
Yes	**-0.18**	**-0.28**	**-0.08**	**0.40**	**0.24**	**0.55**
Physicians or nurses in the family						
No	Ref			Ref		
Yes	**0.21**	**0.05**	**0.37**	-0.04	-0.29	0.21
Experiences of patient safety incidents						
No	Ref			Ref		
Yes	0.18	-0.01	0.36	**-0.30**	**-0.58**	**-0.03**
Survey layout						
Type A	Ref			Ref		
Type B	**-0.32**	**-0.42**	**-0.23**	-0.02	-0.17	0.13
Clarity of medical errors						
Unclear	Ref			Ref		
Clear	**-0.89**	**-0.99**	**-0.80**	**0.93**	**0.78**	**1.08**
Level of harm						
Minor	Ref			Ref		
Major	**-1.85**	**-1.95**	**-1.76**	**6.05**	**5.90**	**6.20**
Disclosure of patient safety incidents						
No disclosure	Ref			Ref		
Full disclosure	**1.24**	**1.15**	**1.34**	**-0.74**	**-0.89**	**-0.59**

CI: confidence interval

^a^after natural log transformation

Similar to the logistic regressions, some variables also showed statistically significant relationships in linear regression. Trust scores were 0.89 points lower (95% CI: -0.99–-0.80) when the medical errors were clear and they were 1.85 points lower (95% CI: -1.95–-1.76) when major harm occurred. The expected amounts of compensation were higher when the medical errors were clear and major harm occurred, than when errors were unclear or when minor harm occurred. Furthermore, participants who had religion gave lower trust scores and expected higher amounts of compensation than those who had not religion.

## Discussion

In this study, we evaluated the effectiveness of DPSI using hypothetical cases in a survey of the Korean general public. The results indicated favorable findings in support of DPSI. The odds of judging the hypothetical cases as medical errors were 0.63 times lower when DPSI was performed than when it was not. The odds of willingness to revisit and recommend a physician were 2.66 and 2.47 times higher, respectively, when patient safety incidents were disclosed than when they were not disclosed. Furthermore, the odds of an intention to file a medical lawsuit were 0.41 times lower and those of an intention to commence criminal proceedings were 0.44 times lower when incidents were disclosed than when they were not disclosed. Trust scores were 1.24 points higher when patient safety incidents were disclosed than when they were not disclosed. Furthermore, the excepted amount of compensation was smaller when patient safety incidents were disclosed than when they were not disclosed.

One of the strengths of this study is the fact that we identified the positive effects of DPSI from a relatively large sample (n = 700). Furthermore, the sample represented the general Korean population considering the comparison results of distribution of age group and gender between the survey participants and national registration population data from the Ministry of Government Administration and Home Affairs. The public preferences regarding DPSI have been well-described in previous studies conducted not only in Korea [3], but also in other countries [11]. This survey quantitatively measured and clarified the positive effects of DPSI, which validated the results of previous studies [18–20]. Several studies described various barriers and obstacle to DPSI, including fear of medical lawsuits and punishment, fear of a damaged professional reputation among colleagues and patients, diminished patient trust, the complexity of the situation, and the absence of a patient safety culture [11, 22]. The findings of this study might be helpful in reducing the concerns of medical professionals about negative effects of DPSI, for example, an increase in legal disputes, a damaged professional reputation among patients, and weakened trust in physicians.

DPSI is not the sole deciding factor involved in judging a situation as a medical error, the willingness to revisit and recommend a physician, the intention to file a lawsuit and commence criminal proceedings, an increase in trust, and determination of the expected amount of compensation. Some variables also showed statistically significant and meaningful relationships in the logistic and linear regressions in this study. For example, trust scores were influenced by the degree of harm and the clarity of medical errors. In addition, the amounts of compensation people were willing to accept showed a greater dependence on the degree of harm and the clarity of medical errors than DPSI. Moreover, our survey revealed that there is no statistically significant correlation between DPSI and judgment on the necessity of an apology. The effectiveness of DPSI might be misrepresented or underrepresented by such findings from the medical professionals’ point of view [3]. When medical professionals are unaccustomed to disclosing patient safety incidents, they tend to resort to past experiences and realize how other factors, such as characteristics of the patient safety incidents, can influence the credibility of physicians and intentions to take legal action. Under such circumstances, medical professionals are less likely to deem DPSI worthwhile. However, it should be noted that DPSI itself proved to be effective even when other factors, such as characteristics of the patient safety incidents or socio-demographics were adjusted. Thus, we can use these findings to promote DPSI for medical professionals who have doubts about its benefits.

Another remarkable result was that participants who had religion tended to set high ethical standards for hypothetical cases. Religious participants gave lower trust scores and expected a higher amount of compensation than those who had not religion. In addition, the odds of the intention to file a medical lawsuit and commence criminal proceedings were higher when participants were religious than when they were not. Although DPSI is an important ethical issue [23], empirical evidence on relationships between DPSI and religion is scarce. Based on our results, we showed that DPSI could be viewed as a more important and inevitable ethical issue for people with religious beliefs.

Although we identified the effects of DPSI on various factors, a comprehensive and system-wide approach will be required to encourage medical professionals to conduct DPSI in real healthcare settings [9]. The creation of a patient safety culture, the introduction of guidelines for DPSI, and education or training for DPSI can facilitate medical professionals to perform DPSI [11]. Furthermore, apology law, which prohibits the use of physicians’ apologies as evidence of their negligence in medical lawsuits, could reduce a medical professional’s reluctance to conduct DPSI [24]. In addition, more empirical evidence of DPSI in real healthcare settings is needed to implement a policy for DPSI in hospitals [11].

This study has some limitations. First, we could only confirm the hypothetical effects of DPSI; we do not know whether the participants will show consistency between hypothetical and actual thoughts and behaviors. Further studies to confirm the impact of a DPSI program or policy in the real world settings should be conducted. In fact, only a very small number of studies have been conducted to monitor the opinions of stakeholders, or to compare situations before and after the adoption of DPSI policy in hospitals. For instance, according to the systematic review [11], there were only 2 previous studies that showed a decline in the number of medical lawsuits and related costs after implementing a DPSI program [25, 26]. Many more studies on real-life hospitals that have adopted a DPSI policy need to be conducted to evaluate its various effects.

Second, the order in which the hypothetical cases were presented seemed to influence a participant’s response. The survey layout showed statistically significant relationships with some outcome variables; however, consistent and specific trends were not observed in the results of logistic and linear regressions. Furthermore, DPSI still showed statistical significance even when the results were adjusted for survey layout. However, future studies that use similar methods to ours should consider the order of presentation of the hypothetical cases.

Third, this study focuses on only 2 types of cases. We examined responses on 8 different hypothetical cases, which varied in terms of whether DPSI was performed or not, the degree of harm, and the clarity of medical errors. However, only 2 types of patient safety incidents were utilized; these were a surgical adverse events case and a medical errors case. Other types of hypothetical cases, such as diagnostic errors, diagnostic delays, and healthcare-associated infection, require further exploration.

## Conclusion

In conclusion, we evaluated the effectiveness of DPSI by surveying 700 members of the general public with 8 hypothetical cases. The results indicated the advantages of DPSI, including a reduction in the likelihood of perceiving a situation as a medical error, promotion of the willingness to revisit and recommend physicians, and discouragement of the intention to commence criminal proceedings against physicians against physicians. Furthermore, DPSI increased patients’ trust in physicians and reduced the expected amount of compensation. This study provides empirical evidence to reduce the psychological burden that DPSI introduction might place on healthcare professionals.

## Supporting information

S1 File Full descriptions of the 8 hypothetical cases in this study. (DOCX)
