# Supplementary material for: Evaluating the expected effects of disclosure of patient safety incidents using hypothetical cases in Korea
Source: PLoS One. 2018 Jun 14;13(6):e0199017. doi: 10.1371/journal.pone.0199017 (PMC6002037; doi:10.1371/journal.pone.0199017)
Supplement: S1 File — (DOCX) [file pone.0199017.s001.docx]

Supporting Information File 1. Full descriptions of the 8 hypothetical cases in this study

Hypothetical case 1.

Sixty-two-year-old *patient A* was hospitalized for surgical treatment of colon cancer diagnosed using colonoscopy. The treating physician, *surgeon B,* successfully performed a laparoscopic colon resection 2 days after admission. However, on the third post-operative day, the patient presented with high fever and tenderness at the surgical site. Imaging tests revealed an abscess at the anastomosis site. *Surgeon* *B* suspected a leak from the suture site. S/he told the patient the truth, expressed sympathy, and apologized, as well as ensuring thorough investigation. A catheter was then inserted for drainage. After a few days, *surgeon B* repeatedly showed sympathy and apologized for the unfortunate outcome, even though no medical error was discovered. Furthermore, s/he promised prevention of adverse events in the future. The patient recovered without reoperation; however he was discharged 10 days later than originally planned.

Hypothetical case 2

Sixty-two-year-old *patient A* was hospitalized for surgical treatment of colon cancer diagnosed using colonoscopy. The treating physician, *surgeon B,* successfully performed a laparoscopic colon resection 2 days after admission. However, on the third post-operative day, the patient presented with high fever and tenderness at the surgical site. Imaging tests revealed an abscess at the anastomosis site. *Surgeon* *B* suspected a leak from suture site. S/he notified the patient about the formation of pus and inserted a catheter for drainage. The patient recovered without reoperation; however, he was discharged 10 days later than originally planned.

Hypothetical case 3

Sixty-two-year-old *patient A* was hospitalized for surgical treatment of colon cancer diagnosed using colonoscopy. The treating physician, *surgeon B,* successfully performed a laparoscopic colon resection 2 days after admission. However, on the third post-operative day, the patient presented with high fever and tenderness at the surgical site. Imaging tests revealed an abscess at the anastomosis site. *Surgeon* *B* suspected a leak from the suture site. S/he told the patient the truth, expressed sympathy, apologized, and ensured thorough investigation. An emergency colostomy was performed on the discharge site. However, the patient developed sepsis and was transferred to the intensive care unit. Unfortunately, the patient died from septic shock on the seventh post-operative day after reoperation. *Surgeon B* repeatedly showed sympathy and apologized for the unfortunate outcome, even though no medical error was discovered. Furthermore, s/he promised prevention of adverse events in the future.

Hypothetical case 4

Sixty-two-year-old *patient A* was hospitalized for surgical treatment of colon cancer diagnosed using colonoscopy. The treating physician, *surgeon B,* successfully performed a laparoscopic colon resection 2 days after admission. However, on the third post-operative day, the patient presented with high fever and tenderness at the surgical site. Imaging tests revealed an abscess at the anastomosis site. *Surgeon* *B* suspected a leak from the suture site. S/he notified the patient about the formation of pus and performed an emergency colostomy on the discharge site. However, the patient developed sepsis and was transferred to the intensive care unit. Unfortunately, the patient expired from septic shock on the seventh post-operative day after the reoperation.

Hypothetical case 5

Fifty-year-old *patient A* was hospitalized because of pneumonia. The patient had a history of penicillin-related anaphylactic shock. Regardless of this fact, the treating physician, *Medical B*, prescribed cephalosporin antibiotics. Luckily, the patient only experienced a mild skin rash. Now, being fully aware of the situation, *medical B* disclosed the truth, expressed sympathy, apologized, and reassured the patient that a thorough investigation would take place. A few days later, *medical B* confessed to mistakes in his/her prescription and delivered an apology. Furthermore, s/he promised the prevention of adverse events in the future. Eventually, the patient fully recovered and was discharged on the tenth day of admission.

Hypothetical case 6

Fifty-year-old *patient A* was hospitalized because of pneumonia. The patient had a medical history of penicillin-related anaphylactic shock. Regardless of this fact, the treating physician, *medical B*, prescribed cephalosporin antibiotics. Luckily, the patient merely experienced a mild skin rash. *Medical B* realized his/her mistake, but chose not to speak to the patient openly about it. Eventually, the patient fully recovered and was discharged on the tenth day of admission.

Hypothetical case 7

Fifty-year-old *patient A* was hospitalized because of pneumonia. The patient had a history of penicillin-related anaphylactic shock. Regardless of this fact, the treating physician, *medical B*, prescribed cephalosporin antibiotics. The patient experienced dyspnea, seizure, decreased consciousness, and hypotension from an anaphylactic reaction. *Medical B* transferred the patient to the intensive care unit for high-level care. After acknowledging the situation, *medical B* disclosed the truth, expressed sympathy, apologized, and assured the patient of a thorough investigation. A few days later, *medical B* confessed mistakes in his/her prescription and delivered an apology. Furthermore, s/he promised the prevention of adverse events in the future. The patient survived, but was left with permanent brain damage from a hypoxic-ischemic injury.

Hypothetical case 8

Fifty-year-old *patient A* was hospitalized because of pneumonia. The patient had a history of penicillin-related anaphylactic shock. Regardless of this fact, the treating physician, *medical B*, prescribed cephalosporin antibiotics. The patient experienced dyspnea, seizure, decreased consciousness, and hypotension from an anaphylactic reaction. *Medical B* transferred the patient to the intensive care unit for high-level care. S/he belatedly recognized the situation, but chose not to disclose the truth to the patient. The patient survived, but was left with permanent brain damage from a hypoxic-ischemic injury.
